# Supplementary material for: Chromothripsis during telomere crisis is independent of NHEJ, and consistent with a replicative origin
Source: Genome Res. 2019 May;29(5):737–49. doi: 10.1101/gr.240705.118 (PMC6499312; doi:10.1101/gr.240705.118)
Supplement: Supplemental Material [file supp_gr.240705.118_Supplemental_file_1.zip › contigs/annotated_contigs/DB57/contig.3.DB57_length_395_mean_cov_4.73164556962.docx]

**DB57_length_395_mean_cov_4.73164556962**

```
TAATTTTTTC|ATATTTTTTAATGTGGATTTTTTAATT|TTACTTTTTTTTGCTC|CAAAGTAGGTGTGTATTTTTGTGGGCTATATGA
           >chr7:89837203-89837230 - E=1e-01 p=0e+00  >chr10:105913085-105913300 - E=6
GATGTTTTGATACAGGCATGCAATGTGAAATAATCACAACATGGAGAATGGGATATTCATCCTCACAAGCATTTATCCTTTGGGTTATA
e-109
AACAATACAGTTACACTCTTCTAGTTATTTTTTAATGTACAGTTAAGTGATTGTTGACTATAGTCACCCTGTTGTGCAATGAAAATTTT

ACC|T|TAACTTGGATCACTTGGTTAAGTAGTGTCTGCCACTTTCTCTATTGTAAAGTTAGTTTTTCCATTTGTAATTTATTAATTTGT
    >chr10:105913814-105913942 + E=1e-65
GGAAAGATACTTTGAGACTATGTAAATATCTTGTTTCTCATCAC
```
